# The Family Affluence Scale as an Indicator for Socioeconomic Status: Validation on Regional Income Differences in the Czech Republic

**DOI:** 10.3390/ijerph14121540

**Published:** 2017-12-08

**Authors:** Vladimir Hobza, Zdenek Hamrik, Jens Bucksch, Bart De Clercq

**Affiliations:** 1 Faculty of Physical Culture, Department of Recreation and Leisure Studies, Palacky University Olomouc, 771 47 Olomouc, Czech Republic; zdenek.hamrik@upol.cz; 2 Department of Natural and Human Sciences, University of Education Heidelberg, D-69032 Heidelberg, Germany; bucksch@ph-heidelberg.de; 3 Department of Public Health, Ghent University, 9000 Gent, Belgium; B.DeClercq@ugent.be

**Keywords:** family affluence, Health Behaviour in School-Aged Children (HBSC), validation, disposable income per household, regional economic differences

## Abstract

The Health Behaviour in School-Aged Children study (HBSC) uses the Family Affluence Scale (FAS) as a tool to identify the socioeconomic status of children and adolescents. Even though it is now widely applied in research studies, the external criterion validation of FAS has not been verified in terms of objective economic indicators in Central Europe. The aim of this study is to validate FAS in terms of disposable income per capita in 14 Czech administrative regions. Regional differences in the FAS score were analyzed using Pearson correlation and linear regression to measure the dependency of the aggregated mean of the FAS index at the regional level on data from the Czech HSBC survey carried out from April to June 2014 (*n* = 10,361). The data analysis has shown an overall positive correlation between the FAS index and regional disposable income (R = 0.77, *p* < 0.01). The regional disposable income per person could explain 59.7% of the variance in the FAS index (*p* < 0.01). By validating individual items, the authors identified three items with a significant correlation (*p* < 0.01): number of computers, dishwasher at home, and number of holidays. FAS seems to be a valid instrument to measure adolescents’ socioeconomic status.

## 1. Introduction

Socioeconomic status has long been studied as a determinant of adult health, and is defined by employment, education and material wealth [1,2]. People with a lower socioeconomic status more commonly suffer from health problems such as heart disease, diabetes, hypertension, and overall mortality [3,4]. Several studies also concluded that parental socioeconomic status was an important factor of their children’s health [5,6]. In recent decades, studies on adolescent health have also tended to focus on the role of social determinants of health (SDH), which are defined by the WHO as “conditions in which people are born, grow, live, work and age. These circumstances are shaped by the distribution of money, power, and resources at global, national and local levels” [7]. It was also confirmed that national wealth is an important factor of adolescent health. However, there are large variations within countries, especially in those with great income inequality. Other factors, such as family affluence and cultural background, also play important roles. This does not only apply within a single country, but seems to apply across countries [8,9]. 

Adolescent health has been continuously monitored by several studies, such as the Global School-Based Student Health survey, or the Health Behaviour in School-Aged Children (HBSC). The HBSC survey uses a continuously developing section of the questionnaire called the Family Affluence Scale (FAS) [10,11,12]. FAS was developed in Scotland as a measure of family affluence. It was discovered that children and adolescents did not have accurate information on their family’s finances, and that a less intrusive, more comprehensible approach had to be utilized to identify their socioeconomic status [10]. The FAS initially contained three items: ownership of a family car, bedroom, and telephone [10]. Some indicators functioned differently due to country specifics, leading to the omission of some questions that were not consistent with socioeconomic status [13]. As more countries joined the HBSC study, FAS required further development (FAS I, FAS II, and FAS III), which incorporated various novel items. The latest FAS III was introduced for the data collection in 2013/2014. 

Due to the widespread use of FAS in child and adolescent health research, it is of high importance to study the validity and reliability of FAS. There are few papers testing the internal validity of FAS [14,15] and external validity of FAS [16,17,18]. Liu et al. [15] tested the validity and reliability of FAS II in Beijing, and concluded that it was a valid and reliable measure of socioeconomic status. Schnohr et al. [19] studied item parameter drift between survey years, and observed the highest item response drift in computers per household. This was thought to be due to a general increase in computer availability. A solution was presented in a paper by Makransky et al. [20] using the Rasch model, which accounts for the shift in probabilities when comparing different survey years. Another study by Currie et al. [11] pointed out that FAS II was no longer able to discriminate between very rich and very poor countries, leading to subsequent validation of a FAS III. Further validation studies used a qualitative approach, in which focus group discussed and analyzed the understanding and meaning of various items among school-aged children [17]. This led to the recommendation of FAS III questions. Another study by Torsheim et al. [18] used psychometric validation of 16 potential indicators of affluence in eight countries, leading to the development of the latest six-item version of FAS III.

However, only a limited number of studies have assessed the external criterion validity of FAS against objective economic criteria such as the gross domestic product (GDP) or national wealth. Moreover, Boyce et al. [16], who confirmed the good criterion validity of FAS through external criterion validation, pointed out that the validity of their study was limited by cultural and social differences between countries and regions. To our knowledge, there is no similar paper that compares FAS between regions, and uses officially available economic data as an external validation criterion in the Central European region. Thus, the study aims to examine the external criterion validity of FAS on regional income differences, using the data from the HBSC 2013/2014 study in the Czech Republic. 

## 2. Methods

### 2.1. Sample

The HBSC study includes 11-, 13- and 15-year-olds school children in representative samples of schools in each of the participating countries. The students respond to a standardised questionnaire during a school lesson after being given instructions by the teacher or survey administrator. The HBSC data on adolescents have been collected every four years since 1985/1986 [11]. 

The last survey in the Czech Republic was conducted between April and June 2014. The data collection was approved by the Ethical Committee of Palacky University, Olomouc (ethical code: 16/2014). The schools were selected randomly after stratification by region and type of school (primary schools and secondary schools). Then, one class from grades 5, 7 and 9 (corresponding to the age categories of 11-, 13- and 15-year-olds) was randomly selected in each school. Out of the 244 contacted schools, 243 schools (99.6%) agreed to participate. The high participation rate could be attributed to the support provided by the Czech Ministry of Health and the Ministry of Education, Youth and Sport.

Data from 14,539 children were obtained. At an individual level, the response rate was 89.2%. The majority of non-response was due to illness or other reasons, for example sports or academic competitions (1729; 10.6%), and 30 children refused to participate in the survey (0.2%). Due to some unlikely responses, missing values throughout the questionnaire, or missing information on age or gender, 364 questionnaires were excluded. Only children aged 11, 13 and 15 years were selected, as defined by the HBSC protocol, leading to a sample of 10,650 children. In 289 cases (2.7%), there were no answers in the FAS section. After the incomplete cases were removed, the final sample was 10,361 respondents.

A detailed description and geographic distribution of the sample is shown in Table 1.

### 2.2. Family Affluence Scale—FAS

FAS III used in the Czech Republic consists of 6 items based on joint assessment and validation from the HBSC FAS development project [17]. The questions include new and refined items from previous FAS versions: Bedrooms (FAS II), Computers (FAS II), Cars (FAS II), Holidays abroad (refined), Dishwasher (new), Bathroom (new), as defined by the HBSC 2013–2014 Protocol. The items and their response categories are as follows:

1. *Does your family own a car or another motorized vehicle?* (No = 0; Yes, one = 1; Yes, two = 2).
2. *Do you have your own bedroom?* (No = 0; Yes = 1).
3. *How many computers (including laptops and tablets, not including game consoles and smartphones) does your family own?* (None = 0, One = 1; Two = 2; More than two = 3).
4. *How many bathrooms (room with a bath/shower or both) are there in your home?* (None = 0; One = 1; Two = 2; More than two = 3).
5. *Does your family have a dishwasher?* (No = 0; Yes = 1).
6. *How many times did you and your family travel out of the Czech Republic for holiday/vacation last year?* (Never = 0; Once = 1; Twice = 2; More than twice = 3).

### 2.3. Macroeconomic Indicator/Validation Criterion

The regional net disposable income of households was selected as the objective economic indicator for family income at the macro level. The net disposable income of households can be defined as the amount that households have available for daily consumption, savings in the form of financial assets, and accumulation of tangible and intangible assets [21]. Disposable income, as a concept, is closer to the idea of income as generally understood in economics, as opposed to national income or gross domestic product (GDP) [22]. The authors decided to use disposable income over regional GDP for a variety of reasons. Firstly, GDP is also influenced by the industry and offices of large companies, which operate in many different regions. However, GDP is attributed to the registered office, which is mostly in Prague, the Czech capital city, which represents a separate region. Secondly, disposable income per household is a more appropriate proxy for family affluence as it shows the average monetary means available for spending in each family. The regional data in Czech Crowns (CZK) per year were provided by the Czech Statistics Office [21] for 2014 (the same year as the HBSC survey), and were standardized across OECD countries. 

### 2.4. Statistical Analyses

The calculation included descriptive statistics and FAS III correlation score, which consists of the following four steps:

1. FAS consists of 6 items. The responses to the items are given as specific values (see Section 2.2 for details) and calculated as an aggregated FAS index ranging from 0 to 13:
  FAS index = (Item 1) + (Item 2) + (Item 3) + (Item 4) + (Item 5) + (Item 6)
  For the purposes of the analysis, the authors decided to use the index value as a continuous variable.
2. The respondents were split by 14 regions. The mean of the FAS index was calculated for each region up to four decimal digits. This is due to the fact that the regional differences are rather subtle. It was tested whether the FAS index was normally distributed, using Kolmogorov–Smirnov tests (0.095; *p* < 0.001). This enabled the use of the arithmetic average as an indicator.
3. Then it was verified whether the differences between FAS scores in various regions were statistically significant using the analysis of variance (ANOVA).
4. Finally, the correlations between the mean regional FAS index and local disposable income per capita were assessed using the Pearson Correlation. The dependences were further analysed by linear regression.

Prague was an outlier value in some of the analyses. Therefore, models with and without Prague were calculated. The following step was an analysis of individual items. As the answer scales of the items are categorical, results were transformed into cumulative probabilities expressing likelihood in a particular region. This was performed in order to use a correlation and regression analysis. For instance, if the responses for “washing machine at home” ranged from 0 = no to 1 = yes, the average score of 0.63 in a particular region can be expressed as 63% likelihood that a household has a dishwasher. As a result, this value can be interpreted as continuous.

Pearson correlations were tested between items to identify possible intercorrelations. This was performed to identify items that provide little unique information. A high intercorrelation between two items means that two answers provide similar information. Thus, both items can be replaced by a single item.

## 3. Results

### 3.1. Regression between FAS and Disposable Income

The mean FAS score in the whole sample was 7.4988 (standard deviation 2.3760), with the highest regional mean in Prague (8.1090), and the lowest level in Pardubice region (7.0297). The initial ANOVA testing identified that the differences in FAS between regions were statistically significant (*F* = 17.776, *p* < 0.01) and thus the mean regional FAS values could be used in the model. 

The average disposable income in the Czech Republic was CZK 202,914 per capita. When calculated by region, the highest disposable income was in Prague (CZK 264,100), whilst the lowest was in Usti nad Labem region (174,662) [21]. For details on FAS and disposable income in all regions, refer to Table 2. 

With all input data available, a Pearson correlation was calculated between the regional FAS index and disposable income. The resulting correlation is significant (*r* = 0.773; π < 0.001). A linear regression analysis suggested that the regional disposable income per person could explain 59.7% of the variance in the FAS index (*p* < 0.001), see Table 3.

In terms of the regions and their position on the regression line, the outlier item is the Prague region, which is the capital city. Even though the disposable income in Prague is significantly higher than in other regions, the FAS index is not as high.

### 3.2. Analyses of FAS Component Items

After confirming that the whole model with the FAS index is significant, a detailed analysis of individual FAS items was performed. The correlation between FAS items and regional disposable income was then calculated. The strongest correlation was identified with item 5 (dishwasher) (*r* = 0.891; π < 0.001), and the weakest correlation with item 2 (own bedroom), which shows no statistical significance (*r* = 0.015; π = 0.958). The mean value for each FAS item can be seen in Table 2, the resulting index of determination in Table 4, correlations and their significance in Table 5.

A regression analysis of individual items concerning disposable income suggested that the Prague region was also an outlier in the model. If Prague was taken out of the model, the R^2^ would change to 54.6%, (*F* = 13.25; *p* < 0.01). This also noticeably changed the regression and significance of some individual questionnaire items. The item “dishwasher at home” yielded the most consistent correlation, regardless of whether the capital city of Prague was included or not (Table 5). Furthermore, the items “family car” and “number of bathrooms” were significant only when Prague was excluded from the statistical model. On the other hand, “family holidays” was significant only with Prague present in the model.

### 3.3. Correlation between Items

The sample was tested to identify the correlation between individual items, to identify which items bring the most unique information to the overall FAS score, and which items could be reduced. Table 6 shows the Spearman correlation of the six FAS items. Although all correlations are statistically significant, the correlation coefficients are quite low, ranging from 0.101 between items 2 (own bedroom) and 3 (computers) to 0.318 between items 1 (car) and 3 (computers).

## 4. Discussion

FAS is commonly used to identify the socioeconomic status of children and adolescents in studies on child health and well-being. It was developed with the HBSC, which has become an international WHO collaborative study with 47 participating countries in Europe and North America. The aim of the paper was to examine the external criterion validity of FAS in terms of the regional wealth indicator. It was observed that FAS and regional disposable income were positively correlated, which confirmed the good external validity of FAS. This finding is in line with a validation study by Boyce et al. [16], which used GDP for an international comparison. The Czech Republic shows relatively low income inequality as expressed by the Gini index: an inequality measure used by the World Bank. The estimated value of the Gini index in 2014 was 25.9, where 0 represents perfect equality, while an index of 100 implies perfect inequality [23]. The Czech regions have a different socioeconomic and historical background. Prague is the most developed region, whereas Moravia-Silesia is traditionally considered an industrial region, and the Usti region tends to be the least developed. This socioeconomic status is also evidenced by the lowest disposable income per capita, and lower than average FAS score. Overall, it was confirmed that FAS has a good external validity in the Czech Republic, and that it could be used to identify the socioeconomic status in children and adolescents.

After confirming that FAS III is a composed index with reliable correlation, the authors further analyzed individual component questions to see their dependency on disposable income. This could suggest individual item validity, as well as possible reasons for some deviations and outliers. One of these outliers is the Prague region for item 2 (Bedroom) and item 4 (Bathrooms), which seem to relate to housing prices. The bedroom item has been shown to be of poor reliability and construct validity in previous studies [14,15]. Prague is also the outlier in item 1 (number of cars), which may be related to developed public transport, or to the fact that Prague is the only region that does not contain rural areas, where daily commuting by car is more common. Another factor in the HBSC study that could influence FAS is the difference in costs of goods and provisions between regions. This makes national comparison more difficult given some of the questionnaire items on FAS. On the other hand, questionnaire item 3 (number of computers) and item 5 (dishwasher at home) seem to be unaffected by location, as Prague is in line with other regions. This may be due to the fact that the prices of computers, dishwashers and holidays show little to no change across regions. As documented in a study on regional prices by Bajgar and Janský [24], the average prices of consumer goods vary in the Czech regions by less than 10%. However, item 6 (number of holidays) is not significant without Prague, which shows both the highest disposable income and highest score in item 6 (number of holidays). This could mean that holidays are mostly perceived as luxury services, correlating strongly with higher disposable income. In the analyses of individual items, it was observed that items 3 (number of computers), 5 (dishwasher at home) and 6 (number of holidays) could be used as a reasonable proxy of family income independently of geographical location. 

In the last section of results, the analytical strength of FAS III items was analyzed by measuring their intercorrelations in the Czech HBSC sample. If two answers are highly correlated, they are likely to cover the same concept (e.g., housing socioeconomic status), and one of them could be eliminated without losing interpretation power. On the other hand, if an item is not correlated, it brings a new aspect of SES not assessed by any other question. The results have shown that the two closest items were cars and number of computers. These two items are similar in the sense that they both indicate purchase of material goods. However, the relatively low correlation shows that each question focuses on a different aspect of the socioeconomic status, and that both items should be included in the survey. The results of an inter-question correlation analysis indicate that each FAS item brings additional independent information, and therefore no item can be easily left out. The correlations with composite FAS scores correspond with the findings by Liu et al. [15].

In line with the present findings, FAS is a suitable tool for the identification of socioeconomic differences. The findings indicate that it is a valid scale corresponding with objective indicators of economic differences among Czech regions. Further development of FAS should include modern trends and consider the impact of outlier regions on the study validity.

### Strengths and Limitations

The strength of the study is the sample size, as it is representative of all Czech regions. The survey is based on international methodology and uses standardised data collection methods. Furthermore, the external criterion validation was performed in one country, which limits the role of cultural and social differences that could compromise international comparisons.

A limitation is the low number (14) of regions in the Czech Republic that were sampled, and the absence of more detailed data on the level of medium-size cities. Some regions are quite heterogeneous and may include more different cities. The paper only uses data from one survey year (2013/2014), which is the latest data available. Using multiple years would require adjustment for item response drift, as identified by Schnohr et al. [19]. In the present study, the authors presumed that the FAS index was a continuous variable. This was a simplification applied due to a large number of questionnaires in regional samples (*n* > 700 per region). The actual FAS value for individual questionnaires is an integer. This also indicates another limitation: the approach does not consider individual cases.

## 5. Conclusions

The analysis of social inequalities in health requires valid and reliable tools to identify socioeconomic status. The HBSC uses continuously developing FAS. The external criterion validation confirmed that the differences in FAS responses per region could be explained well by disposable income per capita. This was statistically significant despite the rather subtle regional differences. Even though the use of regional data is innovative, the findings confirm good validity, as identified by previous validation studies. A practical implication is that FAS is a valid tool, and provides valuable information on the socioeconomic status of children and adolescents. Further development of FAS should consider the size of the region and some well-developed/underdeveloped regions, which may influence the statistical significance and validity of some questionnaire items.

## Figures and Tables

**Table 1:** Sample details.

		Sex		Age (Years)		
Region	Total Respondents (*n*)	Boys (*n*, %)	Girls (*n*, %)	11 (*n*, %)	13 (*n*, %)	15 (*n*, %)
Prague	835	404, 48.4%	431, 51.6%	238, 28.5%	288, 34.5%	309, 37.0%
Central Bohemia	770	381, 49.5%	389, 50.5%	255, 33.1%	251, 32.6%	264, 34.3%
South Bohemia	813	390, 48.0%	423, 52.0%	255, 31.4%	275, 33.8%	283, 34.8%
Plzeň	657	310, 47.2%	347, 52.8%	218, 33.2%	218, 33.2%	221, 33.6%
Karlovy Vary	596	291, 48.8%	305, 51.2%	221, 37.1%	190, 31.9%	185, 31.0%
Ústí nad Labem	693	340, 49.1%	353, 50.9%	207, 29.9%	246, 35.5%	240, 34.6%
Liberec	741	369, 49.8%	372, 50.2%	250, 33.7%	240, 32.4%	251, 33.9%
Hradec Králové	697	351, 50.4%	346, 49.6%	209, 30.0%	219, 31.4%	269, 38.6%
Pardubice	752	373, 49.6%	379, 50.4%	253, 33.6%	247, 32.8%	252, 33.5%
Vysočina	727	362, 49.8%	365, 50.2%	241, 33.2%	229, 31.5%	257, 35.3%
South Moravia	774	383, 49.5%	391, 50.5%	234, 30.2%	278, 35.9%	262, 33.9%
Olomouc	819	402, 49.1%	417, 50.9%	256, 31.3%	263, 32.1%	300, 36.6%
Zlin	722	343, 47.5%	379, 52.5%	223, 30.9%	257, 35.6%	242, 33.5%
Moravia-Silesia	765	394, 51.5%	371, 48.5%	232, 30.3%	272, 35.6%	261, 34.1%
**Total**	**10,361**	**5093, 49.2%**	**5268, 50.8%**	**3292, 31.8%**	**3473, 33.5%**	**3596, 34.7%**

**Table 2:** Regional details of disposable income, Family Affluence Scale (FAS) index, and FAS component items.

Region	Disposable Income	FAS Index	Std. Deviation	Family Car	Own Bedroom	No. of Computers	No. of Bathrooms	Dishwasher in Home	Family Holidays
	(CZK/Year)	Mean (0–13)		Mean (0–2)	Mean (0–1)	Mean (0–3)	Mean (0–3)	Mean (0–1)	Mean (0–3)
Prague	264,100	8.1090	2.3646	1.3873	0.5679	2.5261	1.3583	0.7496	1.7844
Central Bohemia	216,633	7.9662	2.3401	1.5832	0.6667	2.4073	1.4861	0.7063	1.6183
Plzeň	205,083	7.5646	2.4755	1.4984	0.5679	2.2411	1.3222	0.6605	1.6284
South Moravia	203,208	7.5845	2.3741	1.4306	0.616	2.4108	1.3778	0.6517	1.6713
Hradec Králové	198,052	7.3255	2.3201	1.4579	0.5941	2.2442	1.3954	0.6502	1.4634
Vysočina	195,304	7.1183	2.4678	1.5197	0.5759	2.2495	1.459	0.6434	1.5424
Pardubice	193,820	7.0297	2.3404	1.4406	0.5503	2.2585	1.3488	0.6431	1.4397
South Bohemia	193,653	7.3271	2.2867	1.4797	0.5871	2.2576	1.414	0.6645	1.5814
Zlin	189,825	7.2367	2.3199	1.4714	0.5994	2.3472	1.4548	0.6445	1.5503
Liberec	189,176	7.5447	2.2164	1.3525	0.5683	2.1903	1.2939	0.5992	1.5
Moravia-Silesia	184,014	7.7997	2.3226	1.3021	0.5205	2.31	1.3333	0.5837	1.5208
Olomouc	183,173	7.1465	2.3534	1.3548	0.5574	2.2951	1.361	0.563	1.5322
Karlovy Vary	181,819	7.8075	2.3894	1.3993	0.6269	2.2229	1.2607	0.5709	1.5635
Ústí nad Labem	174,662	7.3046	2.3696	1.2746	0.602	2.2266	1.2955	0.5706	1.577

**Table 3:** Regression data between mean regional FAS score and disposable income of households.

Regression Model Coefficients	B	Beta	t	*p*
Constant	5.156		9.258	<0.001
Disposable income	1.179 × 10^−5^	0.773	4.216	0.001
**Regression model summary**	**R**	**R square**	**Std. Error**	
	0.773	0.597	0.2202	
**Regression ANOVA**	**Sum of squares**	**F**	***p***	
Regression	0.862	17.776	0.001	
Residual	0.582			
Total	1.444			

**Table 4:** Index of determination (R^2^) for individual questions.

Region	Family Car	Own Bedroom	No. of Computers	No. of Bathrooms	Dishwasher in Home	Family Holidays	FAS Index	FAS Index
	R^2^	R^2^	R^2^	R^2^	R^2^	R^2^	R^2^	R
All regions	0.076	0.001	0.651	0.038	0.794	0.460	0.773	0.597
Without Prague	0.671	0.159	0.253	0.293	0.797	0.050	0.739	0.548

**Table 5:** Correlation coefficients and significances per questionnaire item including/excluding Prague.

**Correlations—with Prague**						
**Disposable Income of Households**	**Family Car**	**Own Bedroom**	**No. of Computers**	**No. of Bathrooms**	**Dishwasher at Home**	**Family Holiday**
Pearson Correlation	0.275	0.015	0.807 **	0.195	0.891 **	0.678 **
Sig. (2-tailed)	0.342	0.958	0.000	0.504	0.000	0.008
N	14	14	14	14	14	14
**Correlations—without Prague**						
**Disposable Income of Households**	**Family Car**	**Own Bedroom**	**No. of Computers**	**No. of Bathrooms**	**Dishwasher at Home**	**Family Holiday**
Pearson Correlation	0.819 **	0.399	0.503	0.541	0.893 **	0.223
Sig. (2-tailed)	0.001	0.177	0.080	0.056	0.000	0.465
N	13	13	13	13	13	13

** Correlation is significant at the 0.01 level (2-tailed).

**Table 6:** Intercorrelations between item questions (Spearman’s rho).

	Family Car	Own Bedroom	No. of Computers	No. of Bathrooms	Dishwasher in Home	Family Holidays
Family car	1.000	0.159 **	0.318 **	0.307 **	0.281 **	0.235 **
Own bedroom	0.159 **	1.000	0.101 **	0.243 **	0.150 **	0.129 **
No. of computers	0.318 **	0.101 **	1.000	0.204 **	0.257 **	0.183 **
No. of bathrooms	0.307 **	0.243 **	0.204 **	1.000	0.249 **	0.183 **
Dishwasher in home	0.281 **	0.150 **	0.257 **	0.249 **	1.000	0.223 **
Family holidays	0.235 **	0.129 **	0.183 **	0.183 **	0.223 **	1.000

** Correlation is significant at the 0.01 level (2-tailed).
